# Voronoi tessellation as a complement or replacement for confidence ellipses in the visualization of data projection and clustering results

**DOI:** 10.1371/journal.pone.0333653

**Published:** 2026-04-24

**Authors:** Jörn Lötsch, Dario Kringel

**Affiliations:** 1 Institute of Clinical Pharmacology, Goethe - University, Frankfurt am Main, Germany; 2 Fraunhofer Institute for Translational Medicine and Pharmacology ITMP, Frankfurt am Main, Germany; 3 Faculty of Medicine, University of Helsinki, Helsinki, Finland; Julius-Maximilians-Universitat Wurzburg, GERMANY

## Abstract

Visualizing two-dimensional data projections with group-wise coloring and confidence ellipses is a standard approach in biomedical data analysis. However, this method can obscure subtle group overlaps or atypical cases. Voronoi tessellation, which is widely used in crystallography to analyze local structure, offers a parameter-free geometric alternative that can improve the evaluation of group structure in raw or projected data. We implemented Voronoi tessellation as a plot type for two-dimensional biomedical data and compared it with confidence ellipses on three artificial datasets and three biomedical datasets. For datasets with well-separated classes, both visualization techniques effectively delineated groups. Voronoi tessellation more clearly highlighted cases with points overlapping the opposite group, revealed internal group heterogeneity, and enabled quantification of structural discordance via a Voronoi island count as a visualization-intrinsic metric with no equivalent in confidence ellipse approaches. In datasets with moderate or absent group separation, Voronoi tessellation more effectively exposed the lack of meaningful structure, whereas confidence ellipses more clearly indicated distant outliers. Voronoi tessellation also facilitated the identification of clustering failures. Thus, Voronoi tessellation enhances the detection of deviations from expected group patterns and provides geometric insights that complement statistical summaries from confidence ellipses. Therefore, integrating Voronoi tessellation into standard data analysis workflows is a valuable addition for visualizing biomedical data and supports hypothesis validation and exploratory analyses in both raw data visualization and dimensionality reduction or clustering. An R library “VoronoiBiomedPlot” is available at the Comprehensive R Archive Network (CRAN) at https://cran.r-project.org/package=VoronoiBiomedPlot.

## Introduction

Projecting data and inspecting group separation are standard parts of biomedical data analysis workflows. Unsupervised or supervised techniques, such as principal component analysis (PCA [1,2]) or partial least squares discriminant analysis (PLS-DA [3]), respectively, are among commonly used methods to investigate study group separation, with many further methodological alternatives [4]. Consequently, standard software implementations, whether in data science-oriented languages such as R [5] or Python [6], as well as commercial biomedical data analysis packages, offer data projections onto the two-dimensional $R^{\text{2}}$ plane with group-wise coloring of points. The projection of subgroups into specific regions is emphasized via confidence ellipses.

The Voronoi tessellation of the two-dimensional projection plane offers an alternative to confidence ellipses, as we have previously demonstrated in a variety of datasets [4]. Voronoi tessellation is a well-established tool in crystal structure research, where it facilitates the analysis of local atomic arrangements, grain boundaries, and phase transitions [7]. By defining neighbor relations through the identification of shared facets between Voronoi cells, this method provides a parameter-free approach to investigating local structure and nucleation processes in supercooled liquids [8]. Given these capabilities, Voronoi tessellation is well-suited to enhance the (visual) assessment of relationships among individual cases within complex group structures in diverse data sets. Its advantages include quicker visual identification of concentrations of the study group and disruptions to the expected color pattern in the Voronoi tessellation. However, further consideration is needed to determine if Voronoi cell-based projections could replace standard confidence ellipse visualizations and be included as the default in omics analysis software [9]. Therefore, following the successful proof of concept [4], the present report focused on systematic comparison with classical confidence ellipses, along with the introduction of a measure to quantify structural discordance via a Voronoi island count and implementation in an R package.

## Methods

### Confidence ellipses

Confidence ellipses illustrate the uncertainty or variability associated with group means in two dimensions. Mathematically, a confidence ellipse is a two-dimensional generalization of a confidence interval and is typically constructed under the assumption that the data within each group follows a bivariate normal distribution. For a group of projected points with a mean vector μ and a covariance matrix Σ, the confidence ellipse for a specified confidence level (e.g., 95%) is defined as the set of points satisfying the equation


$$(x-\mu {)}^{T}\sum^{-\text{1}}(x-\mu )\leq c$$ (1)

where c is a constant determined by the desired confidence level and related to the quantile of the chi-squared distribution with two degrees of freedom [10].

Geometrically, the confidence ellipse is centered at the group mean, and its axes are aligned with the principal directions of the covariance matrix. The lengths of the axes reflect variability in each direction. A circular ellipse indicates equal variance in both dimensions (uncorrelated variables), while an elongated ellipse indicates higher variance in one direction (correlated variables). The area inside the ellipse represents the region where the true group mean would fall with the specified probability (e.g., 95%) if the experiment were repeated many times.

### Voronoi cells

Voronoi cells [11] are defined as follows. Let $\left\{p_{\text{1}},\;p_{\text{2}},\ldots ,p_{n}\right\}$ be a set of n distinct points in a metric space ${D\subseteq R}^{d}$ with a distance function d(x,y) defined for all x,y∈D. The Voronoi tessellation partitions D into n regions, called Voronoi cells, each associated with a point $p_{i}\in P$.

The Voronoi cell $V_{i}$ corresponding to the point $p_{i}$ is defined as:


$$V_{i}=\left\{x\in D|d\left(x,p_{i}\right)\leq d\left(x,p_{j}\right)\;for\;all\;j\neq i\right\}$$ (2)

The definition of the Voronoi cell $V_{i}$ means that for any point x in the domain D,x will be assigned to the cell of the point $p_{i}$ to which it is closest, according to the distance function d(x,y). Formally, $x\in V_{i}$ if and only if $V_{i}=\left\{d\left(x,p_{i}\right)\leq d\left(x,p_{j}\right)\;for\;all\;j\neq i\right\}$. This ensures that the entire space D is divided into non-overlapping regions, each region containing all points that are closest to a specific center $p_{i}$.

Geometrically, the boundaries between Voronoi cells are formed by the set of points equidistant from two or more centers. In the case of standard Euclidean distance in two dimensions, for example, these boundaries are straight lines, or perpendicular bisectors, between pairs of points. In higher dimensions, the boundaries become hyperplanes. Thus, Voronoi tessellations naturally partition space based on proximity to a given set of points.

### Implementation

The visualization based on Voronoi cell tessellation of the projection plane [4] functions similarly to a “political map”. Specifically, data projections are visualized by plotting data points in a two-dimensional $R^{\text{2}}$ plane. Each point is colored according to its prior classification. Then, Voronoi cells are computed around each data point, and these cells are colored according to the same class labels. Alternatively, the coloring can reflect different classes, such as those resulting from clustering, prior classifications, or study hypotheses.

The resulting plot shows each Voronoi cell colored by class, with the original points overlaid. This allows for an intuitive visual assessment of class separation. Optionally, the cells can be colored according to an alternative classification, such as clustering. This is useful for visually judging the clustering success when contrasting it with prior classification. The code includes two functions. The more complete function, “create_tesselation_plots” produces three types of plots, including confidence ellipses, Voronoi tessellation, or a combination of both, whereas the reduced function, “create_voronoi_plot”, creates only the colored Voronoi cell variant that is the focus of this report. The detailed function call is described in the R code and its description provided at https://github.com/JornLotsch/voronoi_tesselation_plot, with the corresponding archived release on the EU Open Research Repository Zenodo (https://doi.org/10.5281/zenodo.18954701). The main function calls and parameters are also shown in Textbox 1 and Table 1 respectively.

**Table 1:** Function call parameters to invoke various Voronoi tessellations and/or confidence ellipse-style plots of two-dimensional data with (or without) class structure, and list of main outputs of the function. See also https://cran.r-project.org/package=VoronoiBiomedPlot.

Parameter/Output	Type	Default	Description
**Function call parameters**			
**Data**	data.frame	–	Data with ≥2 numeric columns for coordinates
**class_column**	character/vector	NULL	Column name or vector of class labels
**alternative_class_column**	character/vector	NULL	Alternative column name or vector of class labels
**coordinate_columns**	character vector	NULL	Column names to use as coordinates (if NULL, uses first 2 numeric columns)
**case_labels**	character vector	NULL	Individual case labels (uses row numbers if NULL)
**coord_names**	character vector	c(“Dim1”, “Dim2”)	Names for coordinate axes
**Title**	character	NULL	Plot title
**show_labels**	logical	FALSE	Whether to show case labels
**voronoi_alpha**	numeric	0.3	Transparency of Voronoi regions (0–1)
**point_size**	numeric	2	Size of data points
**legend_position**	character/numeric	“bottom”	Legend position
**color_palette**	function/character	NULL	Custom color palette
**add_grid_lines**	logical	FALSE	Whether to add origin grid lines
**color_points**	character	“primary”	Which classification to use for point colors (“primary” or “alternative”)
**fill_voronoi**	character	“primary”	Which classification to use for Voronoi fills (“primary” or “alternative”)
**point_shape**	character	“none”	Which classification to use for Voronoi fills (“primary”, “alternative” or “none”)
**label_fontface**	character	“plain”	Font face for case labels (“plain”, “bold”, “italic”, “bold.italic”)
**label_size**	numeric	3.88	Size of case labels
**show_island_count**	logical	FALSE	Whether to display the Voronoi island count as a plot subtitle
**label_islands_only**	logical	FALSE	Whether to show case labels on plots only for Voronoi islands, for which `show_island_count` must be TRUE
**Outputs**			
**result$scatter_plot**	ggplot		Standard scatter plot of the projected data
**result$voronoi_plot**	ggplot		Voronoi tessellation plot with data points
**result$combined_plot**	ggplot		Combined visualization with additional features

To provide a visualization-intrinsic quantitative measure of class structure disruption, we introduce the Voronoi island count. A Voronoi island is defined as a data point whose Voronoi cell is entirely surrounded by cells belonging to a different class, i.e., every Voronoi neighbor of the point belongs to a class other than its own. This definition is motivated by the observation that such a cell constitutes the strongest possible local signal of class discordance visible in the tessellation: the point is isolated within foreign-class territory. This metric is intrinsic to the tessellation geometry and has no equivalent counterpart in confidence ellipse visualizations, making it a measure of what Voronoi tessellation uniquely adds. Neighbor relationships are determined from the Delaunay triangulation [12] embedded in the “deldir” output (the “dirsgs” component), which is the geometric dual of the Voronoi tessellation: two Voronoi cells share an edge if and only if their corresponding points are connected by a Delaunay edge. Island detection therefore requires no additional computation beyond the tessellation itself. The result is summarized as an island count (absolute number of island cells) and an island rate (proportion of all cells that are islands). When the parameter “show_island_count = TRUE” is passed to the “create_voronoi_plot” function, the island count and rate are displayed as a plot subtitle. The island count is computed using the same classification as determines the Voronoi cell colors (“fill_voronoi” parameter), ensuring the metric is truly visualization-intrinsic and describes the tessellation structure that is actually visible to the viewer. When “fill_voronoi = ‘primary’” (default), islands are identified based on “class_column”; when “fill_voronoi = ‘alternative’”, islands are identified based on “alternative_class_column”. To make the islands more visible, the “label_islands_only” parameter can be toggled to add labels only to cases identified as Voronoi islands.

An R library “VoronoiBiomedPlot” is available at the Comprehensive R Archive Network (CRAN) at https://cran.r-project.org/package=VoronoiBiomedPlot. And can be installed from there or from the GitHub repository (https://github.com/JornLotsch/VoronoiBiomedPlot) via the command “remotes::install_github(“JornLotsch/VoronoiBiomedPlot”)”.

### Data sets

#### Artificial data.

Two-class synthetic benchmark data set: The synthetic dataset comprises nine variables acquired from n = 80 cases from two different classes, $C_{\text{1}}\left(n_{\text{1}}=\text{4}\text{0}\right)$ and C₂ (n₂ = 40). Each observation is represented as a vector in ℝ⁹, corresponding to nine continuous variables with varying degrees of class separation. Variables A and B are sampled from normal distributions with class-specific parameters. For variable A, the distribution within class $C_{i}$ is $A\sim N\left(\mu_{i},\overset{\text{2}}{\underset{i}{\sigma }}\right)$, where $\left(\mu_{\text{1}},\mu_{\text{2}}\right)=(\text{4},\text{8})$ and $\left(\sigma_{\text{1}},\sigma_{\text{2}}\right)=(\text{1},\text{1})$. Variable B is generated using scaled and cross-referenced parameters, i.e., $B\sim N\left({\text{0}.\text{5}\mu }_{\text{2}},\overset{\text{2}}{\underset{\text{2}}{\text{0}.\text{9}\sigma }}\right)$ for class $C_{\text{2}}$ and $B\sim N\left({\text{0}.\text{3}\mu }_{\text{1}},\overset{\text{2}}{\underset{\text{1}}{\text{0}.\text{9}\sigma }}\right)$ for class $C_{\text{1}}$, creating an inverse relationship between classes. Variables E and G are drawn from uniform distributions $E\sim U\left(a_{i},b_{i}\right)$ for each class $C_{i}$, where the bounds are derived from jittered class-specific mean values to ensure class separation. Variables C and D are constructed via weighted sampling from sequential values and subsequent jittering, introducing moderate variability with partial class overlap. Variables H and I are sampled from a common uniform distribution across all classes, with variable I being a jittered linear transformation of H. Data set generation details are available via the R code file “Two_class_artifical_data_example.R” on the report’s public repository.

An alternative version of this dataset was created in which the class labels of three cases in each group were reassigned to the opposite class. This modification introduced controlled misclassification, enabling the evaluation of visualization methods under conditions of label noise and their ability to identify atypical or mislabeled instances within well-separated groups.

Three-class synthetic data sets in a significant and a non-significant variant: The second synthetic data set also comprises nine variables, this time acquired from n = 75 cases from three different classes, $C_{\text{1}}\left(n_{\text{1}}=\text{2}\text{0}\right)$, $C_{\text{2}}\left(n_{\text{2}}=\text{4}\text{0}\right)$ and $C_{\text{3}}\left(n_{\text{3}}=\text{1}\text{5}\right)$. Each observation is again represented as a vector in $R^{\text{9}}$. Variables A and B are sampled from normal distributions with class-specific parameters. For variable A, the distribution within class $C_{i}$ is $A\sim N\left(\mu_{i},\overset{\text{2}}{\underset{i}{\sigma }}\right)$, where $\left(\mu_{\text{1}},\mu_{\text{2}},\mu_{\text{3}}\right)=(\text{4},\text{6},\text{8})$ and $\left(\sigma_{\text{1}},\sigma_{\text{2}},\sigma_{\text{3}}\right)=(\text{2},\text{4},\text{3})$. Variable B is generated analogously, using scaled versions of the same parameters. Variables E and G are drawn from uniform distributions $E\sim U\left(a_{i},b_{i}\right)$ for each class $C_{i}$. Variables C and D are constructed via weighted and jittered sampling from uniform distributions, introducing moderate variability and partial class overlap. Variables H and I are sampled from a common uniform distribution across all classes with minimal class discrimination. In the present random realization of the data set, group differences happened to be significant at p < 0.05, according to Kruskal-Wallis tests [13], for variables A, B, E and G. Data set generation details are available via the R code file “Three_class_artifical_data_example.R” on the report’s public repository.

An alternative version of the above data set without significant group differences (all Kruskal-Wallis tests: p > 0.05) was generated by independently permuting each variable in the original dataset, thus destroying the association between the variables and class membership.

Clustering problem data set: The data set used to illustrate the limitations of clustering algorithms such as k-means was taken from the Fundamental Clustering Problems Suite (FCPS), a benchmark collection designed to expose common challenges encountered in real-world clustering tasks [14]. The FCPS suite includes artificial datasets constructed to highlight specific structural difficulties for clustering algorithms, such as varying cluster shapes, densities, and separations. Selected for this study, the two-dimensional “Lsun” dataset comprises 400 samples distributed across three well-separated classes. These classes differ markedly in their geometric properties: one forms a roughly spherical cluster, while the other two are rectangular (“brick-shaped”) and vary in size. This configuration introduces varying cluster variances and densities, presenting a significant challenge for algorithms like k-means [15,16], which assumes that clusters are hyperspheric and therefore perform poorly on the “Lsun” dataset [17]. The clustering demonstration was done using the “kmeans” function from the R “stats” core package, setting the parameters centers = 3 (the original class number in the “Lsun” dataset) and nstart = 100; for details, see the R code file “Lsun_example.R” on the report’s permanent public repository.

#### Biomedical data.

Sensitivity to experimental nociceptive stimuli (“QSTpainEJPtransf”): This psychophysical pain-related dataset (“QSTpainEJP” [18]) was available from an in-house study on clinical quantitative sensory testing (QST) involving 127 healthy subjects [19]. The data set includes 22 sensory measures of pain, i.e., nine from classical pain models (e.g., pressure, cold, electric, chemical and laser-evoked pain thresholds and intensities), and ten from a clinically established QST battery [20]. The QST parameters encompass a range of thermal and mechanical pain and sensation thresholds. Measures were harmonized to ensure that higher values indicate greater pain and were log-transformed as appropriate. Detailed protocols and variable descriptions are available in the original publications.

For the present purpose of data visualization, only the n = 72 complete cases were projected to avoid imputation of missing’s (34 men and 38 women). Since sex differences in pain are a widely reported observation [21,22], sex was used as the data set’s class structure for the present purpose (coding: 1 = male, 2 = female). For details, see the R code “pain_data_example.R” on the report’s public repository.

Clinical lipidomics of rheumatic diseases (“PsA_lipidomics”): This lipidomics dataset (“PsA_lipidomics” [23]; https://doi.org/10.17632/32xts2zxdc.1) includes plasma lipid profiles from 81 patients diagnosed with psoriatic arthritis (PsA) and 26 healthy control subjects matched by age and sex. This results in a data matrix of 107 subjects and 292 lipid markers [24]. Samples were collected as part of a cross-sectional study at a university-based tertiary care rheumatology center. Both targeted and untargeted assays were employed to capture a broad spectrum of lipid classes, including carnitines, ceramides, glycerophospholipids, sphingolipids, glycerolipids, fatty acids, sterols, esters, and endocannabinoids.

During analysis, a subset of control samples was observed to cluster anomalously with PsA patients. Further investigation revealed that these outliers were due to batch effects; control samples processed in separate batches exhibited divergent data projections, indicating insufficient normalization and batch correction. These characteristics made the dataset suitable for comparing standard confidence ellipse plots with the proposed Voronoi tessellation approach, with a focus on identifying and interpreting problematic or mislabeled cases. Additional methodological details can be found at https://data.mendeley.com/datasets/9v8ndhctvz/1. The data set’s processing for this report was similar to that of the above pain-related data set (see R code “PsA_data_example.R” on the report’s public repository.

COVID-19 metabolomics data set (“covid_metabolomics”): Another example was processed using the MetaboAnalyst 6.0 web-based platform [9], which is a comprehensive tool for metabolomics data analysis available at https://www.metaboanalyst.ca/. Specifically, an online statistical analysis was performed at https://dev.metaboanalyst.ca/MetaboAnalyst/upload/MultifacUploadView.xhtml on liquid chromatography-mass spectrometry (LC-MS) peak intensity data from 59 samples, including 20 healthy controls and 39 patients with SARS-CoV-2 infection (data from https://api2.xialab.ca/api/download/metaboanalyst/covid_metabolomics_data.csv, metadata from https://api2.xialab.ca/api/download/metaboanalyst/covid_metadata_multiclass.csv).

This resulted in a data matrix of 59 samples by 2,054 peaks (m/z and retention time). Four metadata factors were included: Diagnosis, Gender, Treatment, and Age. Data preprocessing involved variance filtering using a nonparametric relative standard deviation (median absolute deviation normalized by median), abundance filtering based on median intensity values, median normalization, and autoscaling (mean-centering followed by division by the standard deviation of each variable). The platform’s interactive PCA was conducted, and the resulting loading data was downloaded and plotted for the present analysis.

### Experimentation

Coding was done in the R language (Ihaka and Gentleman 1996) using the R software package (R Development Core Team 2008), version 4.5.0 for Linux, with the PyCharm integrated development environment (version 2025.1.1.1; Professional Edition; JetBrains c.r.o., Prague, Czech Republic), which provides an AI Assistant plugin (https://plugins.jetbrains.com/plugin/22282-jetbrains-ai-assistant, version 251.26094.80.11 for original coding, and 253.29346.420 for the revision of the manuscript), which was used on own previous code [4]. The R code uses the “deldir” R package (https://cran.r-project.org/package=deldir [25]) to compute the Voronoi tessellation of a set of two-dimensional points and assign each cell to a class. This is a novel replacement for the now-obsolete R package previously used [4], so the present code reintroduces easy generation of Voronoi tessellations for biomedical researchers. The R package “ggplot2” (https://cran.r-project.org/package=ggplot2 [26]) was used for plotting, with a colorblind-friendly palette originally taken from the “ggthemes” R package [27]).

The Voronoi tessellation-based visualization method was tested on the datasets mentioned above. Experiments included plotting the raw data for low-dimensional datasets, such as the artificial FCPS::LSun dataset (see below), or after applying a few standard projection techniques, including principal component analysis (PCA [1,2]), partial least squares discriminant analysis (PLS-DA [3]), and occasionally Uniform Manifold Approximation and Projection (UMAP [28]). PCA and PLS-DA were implemented using the R package “mixOmics” (https://www.bioconductor.org/packages/release/bioc/html/mixOmics.html [29]), and UMAP was implemented using the R package “umap” (https://cran.r-project.org/package=umap [30]) with default parameters. In addition, clustering was performed on either unprojected (“LSun”, see below) or projected data, using k-means (with the parameter nstart = 100) and single-linkage hierarchical clustering as examples, chosen based on prior experience with some of the datasets in previous work such as [17].

## Results

### Visualization of clear class structures

**Fig 1:**
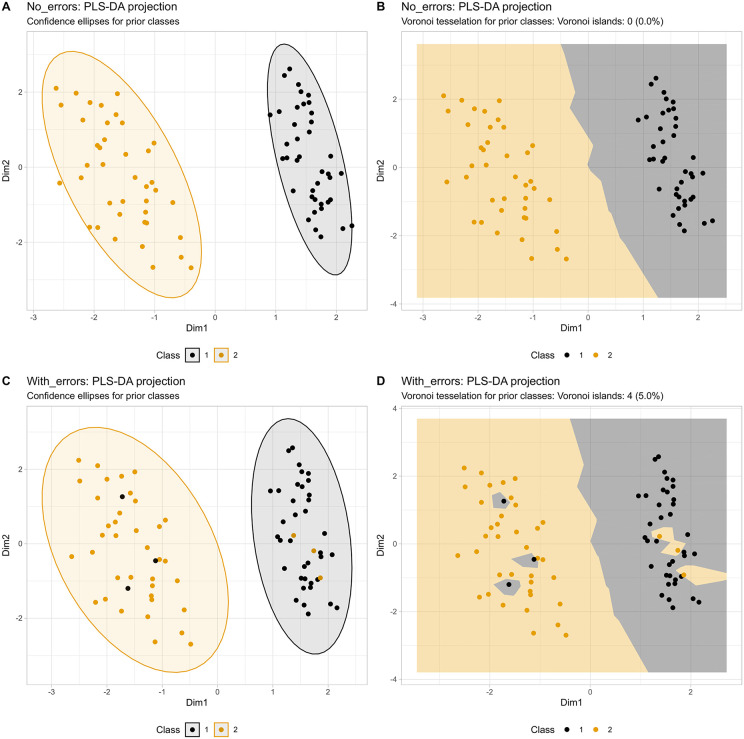
PLS-DA projection results for a nine-dimensional, two-class synthetic benchmark dataset (${𝐧}_{1,2}=40/40$). **A**: Standard 95% confidence ellipse visualization on the ${𝐑}^{\text{2}}$ plane with color coding for the original (prior) classes. **B**: The same projection as in panel A, but with Voronoi tessellation instead of confidence ellipses. **C** and **D**: Introduction of class aberrations: Three instances per class were assigned to the opposite class, after which the PLS-DA projection was repeated. This resulted in only minor changes to the projection. However, panels C and D demonstrate the visualization of the aberrant cases, which are almost hidden in C and very clear in **D**.

In data sets with two classes that are clearly separated on the $R^{\text{2}}$ plane of a PLS-DA projection (Fig 1), visualization using confidence ellipses is adequate (Fig 1A). Notably, the Voronoi tessellation provides an equally clear alternative (Fig 1B), supporting its suitability as a visualization method comparable to the current standard. However, when three points per class are switched to the respective opposite class, the irregularity may escape attention in the ellipses plot (Fig 1C), but it is clearly evident in the Voronoi tessellated plot (Fig 1D).

The Voronoi island count quantifies this observation: in the label-switched dataset (Fig 1D), four islands were detected (island rate: 5.0%). The remaining two switched cases, although visually discordant, retained at least one same-class Voronoi neighbor due to their proximity to the group boundary and therefore do not qualify as islands. This distinction is informative: the tessellation correctly differentiates between fully enclosed discordant cases, which is the strongest signal, and boundary-adjacent discordant cases, which are visible in the plot but represent a weaker form of discordance. The island count thus complements visual inspection rather than replacing it.

### Performance in complex and ambiguous separation scenarios

**Fig 2:**
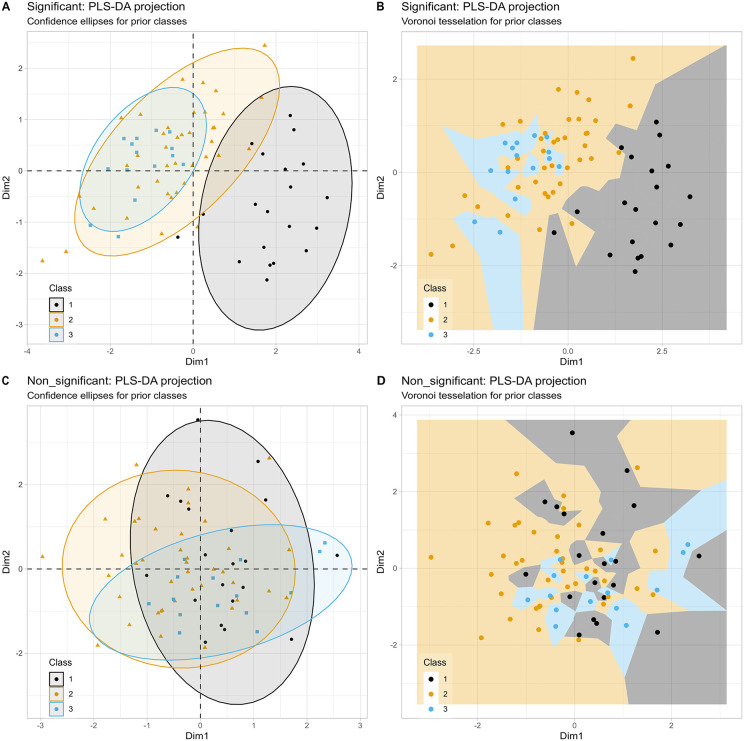
PLS-DA projection for a nine-dimensional, three-class synthetic dataset (${𝐧}_{1,2,3}=20/40/15$) with significant and non-significant variants. **A**: Standard 95% confidence ellipse visualization on the ${𝐑}^{\text{2}}$ plane, with color coding for the original classes in the significant data scenario. **B**: The same projection as in panel A, but with Voronoi tessellation instead of confidence ellipses. C and D: Results of PLS-DA projection following randomly permuting each variable to make the class differences non-significant.

In more complex scenarios where three groups differ to varying degrees across several variables, both Voronoi tessellation and confidence ellipses provide consistent visualizations (Fig 2A and B). The tessellated plot (Fig 2B) may reveal slightly more pronounced group segregation, but it does not clearly outperform the confidence ellipse plot in clarity. However, when the dataset is permuted, confidence ellipses become less decisive in indicating the now absent significant group segregation (Fig 2C and D). This leaves both segregated and non-segregated group structures as possible interpretations (Fig 2C). In contrast, Voronoi tessellation clearly shows the lack of meaningful group segregation in the permuted data. The cells disperse across the $R^{\text{2}}$ plane without any evident concentration or pattern (Fig 2D). Thus, Voronoi tessellation appears superior in distinctly conveying the absence of group structure in ambiguous cases.

### Detection of Voronoi islands and outliers in biomedical data

**Fig 3:**
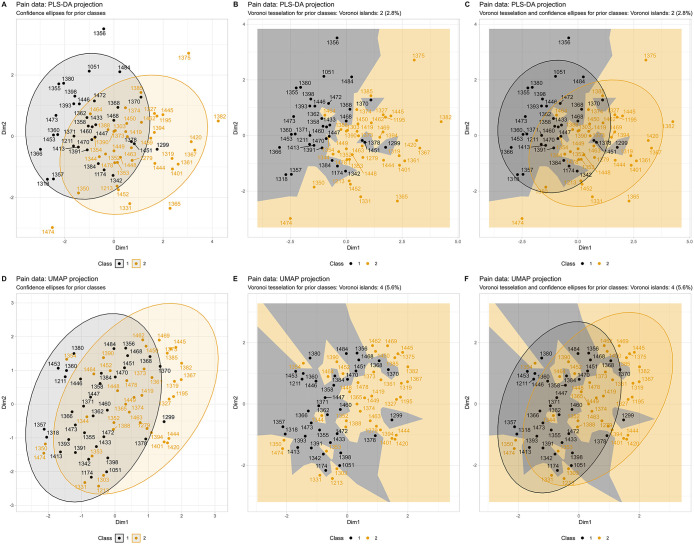
PLS-DA and UMAP projection results for quantitative sensory testing (QST) pain data from 72 healthy subjects (34 men, 38 women) across 19 pain-related variables (dataset “QSTpainEJP”). Individual cases are labeled to enable follow-up analysis at the single-case level. **A:** Standard confidence ellipses on the ${𝐑}^{\text{2}}$ plane with color coding for sex groups. **B:** shows the same projection as panel A, but with Voronoi tessellation instead of confidence ellipses. **C:** Combined visualization of both approaches. **D – F**: Similar visualization as in panels A – C, but with UMAP projection.

A more detailed visualization of group structure is achieved using Voronoi tessellation in the biomedical, pain-related “QSTpainEJP” data set (Fig 3). The separation of groups defined by sex is evident in both displays of the PLS-DA projection (Fig 3 top row of panels). Standard outliers, such as cases #1356, 1365, 1375, 1382, and 1474, can be easily identified when case labeling is enabled (see Textbox 1), as they are significantly distant from the primary clusters. In the Voronoi tessellation, additional cases (#1299, 1378, and 1451) appear as Voronoi islands: their cells are entirely enclosed within the opposite sex’s territory, with every Voronoi neighbor belonging to the other group. Unlike standard outliers that are simply distant from all clusters, Voronoi islands signal class discordance within the interior of the projection and may indicate data or laboratory errors, novel phenotypes, or unconsidered conditions, necessitating further investigation. The alternative UMAP projection provided a less distinct separation of the pain data by sex (Fig 3, bottom row of panels). This further emphasizes the utility of the present visualization method for obtaining a quick and intuitive overview of a given data projection and its associated class separation. That is, the scattered, interleaved Voronoi cell pattern in the UMAP panels is itself a diagnostic signal, indicating that the projection does not strongly support the posited class structure. The Voronoi island count provides a quantitative summary of this pattern: 2 islands (2.8%) were identified in the PLS-DA projection (Fig 3 top row of panels), corresponding to cases whose cells were entirely enclosed within the opposite sex’s territory. In the UMAP projection, which provided weaker overall sex-based separation, the island count increased to 4 (5.6%), consistent with the greater intermixing of cell territories visible in panels (Fig 3D–F).

**Fig 4:**
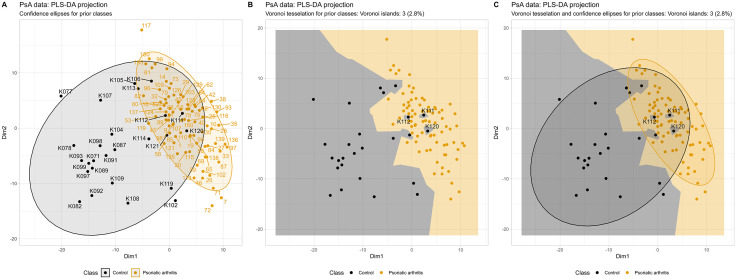
PLS-DA projection results for lipidomic data from 81 patients with psoriatic arthritis (PsA) and 26 healthy controls across 292 lipid markers (dataset “PsA_lipidomics”). Individual cases are labeled to enable follow-up analysis at the single-case level. **A:** Standard confidence ellipses on the ${𝐑}^{\text{2}}$ plane with color coding for diagnostic groups. **B:** The same projection as panel A, but with Voronoi tessellation. The controls (ID code starting with “K”) projected among PsA patients (grey spots within a yellow zone) are among cases with failed laboratory batch correction. **C:** Combined visualization of both approaches. The Voronoi tessellation plots demonstrate the effect of the parameter “label_islands_only”.

A similar pattern emerged in the analysis of the “PsA_lipidomics” dataset, where PLS-DA projections revealed irregularities in the group structure due to batch effects (Fig 4). Notably, several PsA patient samples were scattered among the healthy controls, while most patients clustered together as expected. The Voronoi tessellation more clearly highlighted this dispersion than confidence ellipse visualizations did, and three Voronoi islands (2.8%) were identified: control samples whose cells were entirely enclosed within PsA patient territory. Controls such as #K112, K111, and K120 projected at the periphery of their group or in proximity to patient samples and corresponded to cases with known batch normalization issues. As these cases were subsequently attributed to batch effects rather than true biological similarity with patients, the island count here reflects a data quality signal rather than a biological finding, illustrating that the same metric can flag both types of discordance depending on context.

**Fig 5:**
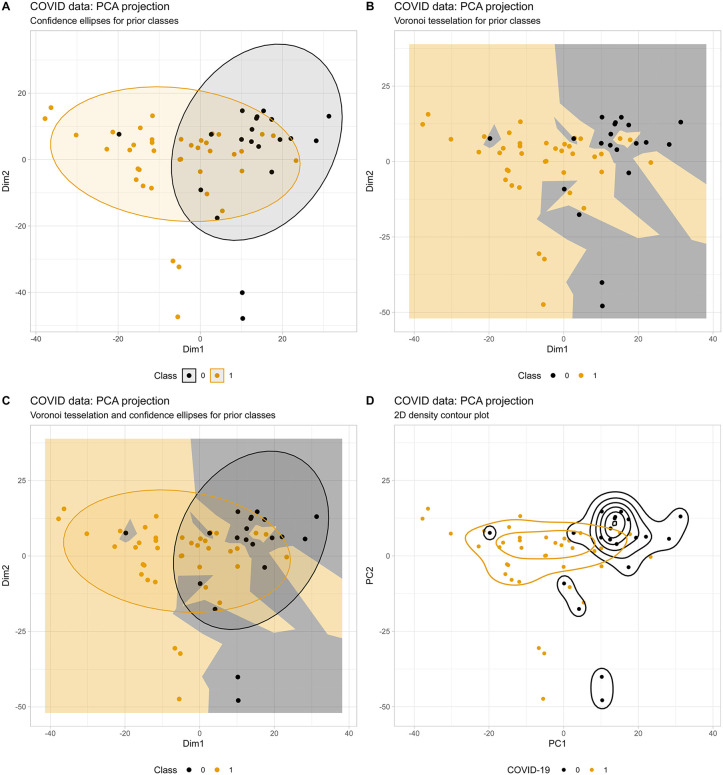
PCA projection results for metabolomics data from 39 COVID-19 patients and 20 healthy controls across metabolite markers (dataset “covid_metabolomics”). Individual cases are labeled to enable follow-up analysis at the single-case level. The data processing and PCA had been performed directly at the MetaboAnalyst 6.0 web site at https://www.metaboanalyst.ca/. **A:** Standard confidence ellipses on the ${𝐑}^{\text{2}}$ plane with color coding for diagnostic groups. **B:** The same projection as panel A, but with Voronoi tessellation (island count omitted as an option in the R library). The tessellation reveals clear spatial separation between COVID-19 patients and healthy controls, however, with overlap between groups. **C:** Combined visualization of both approaches. **D:** Dot plot overlaid with a 2D density contour plot analogous to the MetaboAnalyst 6.0 [9] default.

The “covid_metabolomics” dataset further demonstrated the advantages of Voronoi tessellation over standard visualization techniques for analyzing the effects of SARS-CoV-2 on metabolism (Fig 5). Although group separation between patients with and without the disease was apparent in the PCA projection, the Voronoi tessellation provided clearer identification of outliers and Voronoi islands whose projections overlapped with the opposite class. These observations are consistent with those from other biomedical datasets. Please also compare the default plot style from the “MetaboAnalyst” platform in Fig 5D. It should also be noted that the analysis of this dataset was primarily conducted to create a plot suitable for the current presentation. To generate scientifically valid results on the metabolomic pattern of patients with COVID-19, the data processing steps and resulting projections may differ.

### Class structure detection and agreement analysis

**Fig 6:**
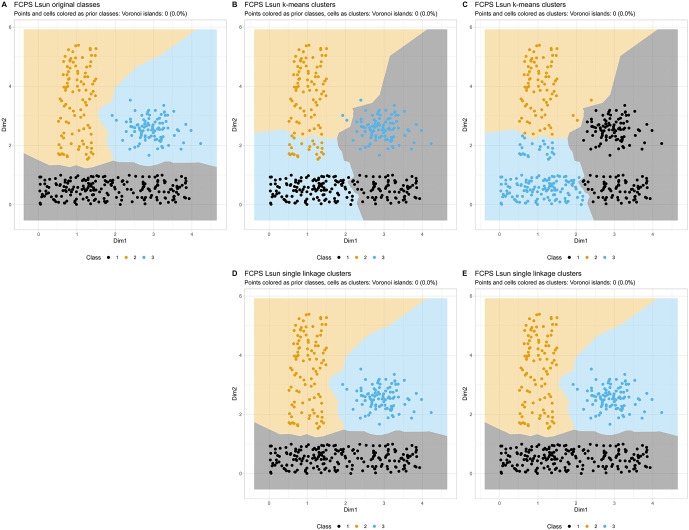
Example of intuitive visualization of clustering quality. Voronoi tessellation of a 2d plot of the original (non-projected) FCPS [14] artificial “Lsun” dataset comparing original class structure with a non-working clustering solution (k-means; k = 3), and a working clustering solution (hierarchical clustering with single linkage). **A:** Original class structure, with points and cells colored according to prior classes. **B:** Comparative visualization in which the points are colored according to the original classes and the Voronoi cells are colored according to the k-means cluster assignments, showing the agreement and disagreement between the prior classification and the unsupervised clustering. **C:** k-means clustering results, with both points and Voronoi cells colored according to cluster assignments. **D** and **E:** As B and C, but with the hierarchical single linkage clustering.

Voronoi tessellation also proved useful for visualizing class or cluster structures and highlighting clustering failures. For example, the “Lsun” dataset, constructed with two rectangular clusters, typically defies correct clustering by the k-means algorithm, as previously shown [14,17]. The Voronoi tessellation plot improves visibility of the original class structure (Fig 6A). As expected, k-means clustering failed to capture this structure, instead producing clusters that corresponded better to its inherent assumption of circular shapes in this two-dimensional dataset. By switching the coloring of data points and Voronoi cells between true classes and clusters using different combinations of the function parameters (“class_column”, “alternative_class_column”, “color_points”, “fill_voronoi”; see Textbox 1), the incorrect clustering became clearly visible (Fig 6B and C). For comparison, single linkage hierarchical clustering was applied, which can handle this dataset and produced a successful clustering solution (Fig 6D and E). The difference between the failed and satisfactory clustering became evident in the Voronoi tessellation plots.

## Discussion

The strength of Voronoi tessellation, whether applied to data projection planes or raw 2D coordinates, lies in its ability to highlight disruptions in expected patterns. This qualifies it as the method of choice in crystallography [7,8], and as the current demonstrations indicate, it appears to qualify for omics research as well. The tessellation of the projection plane makes it easier to identify data points that deviate from their fellow class members with respect to the main projection direction. The visualization effectively emphasizes what might be termed “internal outliers”. Conversely, conventional (“external”) outliers may be less visually prominent in this framework, though they can still be spotted easily, although the definition of outliers can vary [31]. However, combining Voronoi tessellations with confidence ellipses may be a complementary visualization strategy offering statistical and geometric perspectives on data clustering. Confidence ellipses reveal distributional characteristics. Voronoi diagrams, on the other hand, provide an intuitive representation of decision boundaries based on nearest-neighbor proximity. This dual approach leverages the strengths of both methods to offer a more comprehensive view of group structure and enhanced outlier detection capabilities.

Voronoi diagrams divide space into regions based on proximity to a set of points, which gives a clear visual partition of influence or dominance regions, which is ideal for spatial or clustered data, such as ecological zones, cellular territories, or geographical mapping [32]. Voronoi cells inherently define local neighborhoods based on distance, unlike PCA or PLS-DA plots, which may distort neighborhood relationships due to projection into lower-dimensional space, this is especially helpful in data where local context matters, like tissue analysis in histology or population distribution. [33,34]. Despite that, PCA and PLS-DA compress high-dimensional data into 2 or 3 dimensions, which can lead to loss of important variance or discriminative features. Voronoi tessellation can work directly with 2D spatial data without such compression, preserving more truthful geometrical relationships [35–37].

Incorporating Voronoi tessellation is an efficient approach to visualizing class boundaries in projected data spaces. However, it is not limited to projected data, as demonstrated by the “Lsun” data set example where clustering was done on the non-projected data. Unlike traditional clustering visualizations, which rely solely on point distributions, Voronoi regions provide explicit territorial boundaries for each observation. This creates an intuitive classification map of the feature space, which facilitates identifying potential misclassification zones, interpreting the local neighborhood structure around individual data points, and detecting regions of class overlap that may not be apparent in conventional scatter plots. That is, the visualization provides a diagnostic signal that points to data set instances that deviate in their projection from the expectations based on group membership. Whether these deviations are due to laboratory or data errors, represent a novel finding, or merely express normal data variation, is then subject to interpretation by the research team. The visualization merely helps identify cases for investigation.

Voronoi tessellations are especially useful in semi-supervised settings where they enable comparisons between data projections and prior classifications. This includes assessing whether the projected data supports existing classifications. Discrepancies between clustering results and the underlying structure can be easily identified through visual inspection. For instance, as illustrated in the “Lsun” example (Fig 6C), a solitary grey cluster on the right appears as two dense regions upon examination using Voronoi tessellation. This highlights shortcomings in the actual clustering approach. For numerical evaluation of cluster quality, standard metrics can be calculated independently from the present visualization technique, such as cluster accuracy, the (adjusted) Rand index [38], or others. This remains independent of the current visualization and beyond its scope, while in the present context a visualization-intrinsic quantitative measure is provided with the Voronoi island count.

### Interpretation of Voronoi-discordant cases

When a data point’s Voronoi cell is colored differently from the point itself, indicating that the point projects into a region dominated by a different class, the researcher faces an interpretive decision. Three scenarios should be considered.

First, technical or data-quality issues should be excluded. Batch effects, sample labeling errors, preprocessing failures, or instrument drift can all cause individual samples to project anomalously. A discordant case that co-occurs with a known batch boundary or a sample flagged during quality control should be investigated via data provenance review before any biological interpretation is attempted. The “PsA_lipidomics” dataset (Fig 4) illustrates this scenario: the discordant control samples corresponded to samples processed in a separate batch with insufficient normalization. Second, if technical explanations are ruled out, the case may represent a genuinely atypical biological instance: a novel phenotype, a subpopulation not anticipated by the original study design, a mixed diagnosis, or a case in transition between states. Such cases merit targeted follow-up analysis, such as re-examination of raw measurements, additional clinical variables, or independent replication. The pain dataset (Fig 3) provides examples where discordant cases may reflect genuine biological variability in pain sensitivity that cuts across the sex-based classification. Third, in datasets where groups naturally overlap or the class boundary is diffuse, a degree of Voronoi discordance is expected and does not indicate an error or a novel finding. When group separation is weak or the projection collapses a continuous gradient into a discrete classification, some cells near the boundary will inevitably project into foreign-class territory. In such cases, the overall pattern of discordance, i.e., its spatial distribution and prevalence, as quantified by the island rate, is more informative than any individual discordant case.

Voronoi islands, in which all neighboring cells belong to a different class, constitute the most extreme form of discordance and should receive the highest investigative priority, as they are least likely to arise from boundary overlap alone. Not every label-discordant case forms an isolated island: a switched case located near a group boundary may still share a Voronoi edge with a same-class instance, and two points projected among opposite classes but positioned closely together will each share at least one edge with a member of their own class, as illustrated in the right gray region of Fig 1D. This is not a limitation of the visualization. The tessellation correctly differentiates between fully enclosed discordant cases, which represent the strongest signal, and boundary-adjacent discordant cases, which are visible in the plot but represent a weaker form of discordance. The Voronoi island count provides a scalar summary of the strongest class-discordance signal across the entire dataset. Importantly, visualization serves as a tool for judging results and generating hypotheses, not as a classifier itself. Identifying discordant cases marks the beginning of an interpretive process, with appropriate follow-up depending on the biological question, data quality, and possibly projection strength. The Voronoi islands enhance the concept of “looking at the errors,” i.e., data instances that are apparently located in the “wrong place.” Whether or not these instances are actually errors must be determined subsequently, once the attention has been drawn to them.

### Strengths and limitations

In the actual implementation, support for dual classification systems (primary and alternative; see Textbox 1) addresses a common challenge in multivariate analysis, where data points may belong to multiple taxonomic or functional categories simultaneously. This flexibility enables researchers to explore different classification schemes within the same projected space and compare how different grouping strategies affect visual separation patterns.

Case labeling, enabled via the “show_labels” parameter, serves different purposes depending on the context of use. In exploratory or interactive analysis, enabling labels for all cases allows rapid identification of specific data points for follow-up. In publication figures, however, labeling all cases in high-density datasets can produce cluttered displays. For such contexts, we recommend a selective labeling strategy: the “case_labels “argument accepts a vector in which uninformative positions are set to empty strings, suppressing those labels while retaining “ggrepel”-based collision avoidance for the labeled subset (see https://cran.r-project.org/package=ggrepel). The suitability of case labeling depends on the dataset size and the width and height of the exported figure. Therefore, the R implementation provides the “show_labels” parameter to toggle case labeling as needed, and the “show_island_count” subtitle provides a quantitative summary that reduces the need for exhaustive individual labeling, or can be combined with the parameter “label_islands_only = TRUE” which reduces the labeling to cases identified as Voronoi islands (for example, see Fig 4).

A characteristic of the Voronoi island metric is that it depends on local neighbor topology, not global visual impression. If two or more points from the same class are located within a region dominated by the opposite class but happen to be adjacent to each other (sharing a Voronoi edge), they will not be counted as islands because they each have at least one same-class neighbor. This is correct as intended, but this behavior emphasizes that the island count is a geometric measure of cell-level isolation, not a general measure of spatial segregation. Consequently, the island count should be interpreted in conjunction with visual inspection of the tessellation plot itself, rather than as a standalone summary statistic. The metric quantifies a specific geometric pattern, i.e., complete local isolation, but does not capture all forms of class structure disruption visible in the tessellation. In principle, the concept could be extended to define more general neighborhood-based metrics that also account for small same-class clusters embedded within regions of another class; however, the development and systematic evaluation of such extensions lie beyond the scope of the present work, which focuses on introducing a data visualization alternative to confidence ellipses.

While this visualization approach provides insights into the class structure of a data set, the effectiveness of the method depends on the quality of the initial dimensionality reduction. Poor projection techniques may obscure rather than reveal class relationships [39]. Therefore, choosing the right projection techniques remains a key component of the data analysis workflow. Although each data set is unique, one can rely on available comparative benchmarking results [4,17], although *a priori* definition of the best projection method, including “none”, is difficult. The appearance of the tessellation (dispersed, interleaved cells versus compact, segregated regions) is itself informative about projection quality and the degree of class separation supported by the data. The increase in island rate from 2.8% under PLS-DA to 5.6% under UMAP for the same dataset further illustrates this: the metric is sensitive not only to the presence of atypical cases but also to the overall quality of the projection. Additionally, the Voronoi tessellation approach assumes that proximity in the reduced space corresponds to similarity in the original, high-dimensional space, which is not always the case.

## Conclusions

This study introduces a visualization framework integrating Voronoi tessellation and optional confidence ellipses to improve the evaluation of data projections and clustering results. Across artificial and biomedical datasets, the method demonstrated superior sensitivity in detecting structural inconsistencies between projected data and known classifications. It outperformed conventional scatter plots with confidence ellipses alone. Notably, the method enabled clearer identification of cases in which the clustering results did not align with the underlying group structures. These findings establish Voronoi tessellation as a robust alternative to standard confidence ellipse visualizations in omics analysis software and highlight the added value of a combined visualization strategy. Overall, the Voronoi‑tessellation-based visualizations of data point projections in the context of class membership provide a practical and effective tool for more reliable interpretation of dimensionality reduction and clustering in various data analysis applications.

Textbox 1: R function calls for the 2D data visualization with Voronoi-tessellation. For parameter definitions, see Table 1 and at the report’s public code repository (see Data availability statement).

1. results <-

2. create_tesselation_plots(

3. data,

4. class_column = NULL,

5. alternative_class_column = NULL,

6. coordinate_columns = NULL,

7. case_labels = NULL,

8. coord_names = c("Dim1", "Dim2"),

9. title = NULL,

10. show_labels = FALSE,

11. ellipse_alpha = 0.1,

12. voronoi_alpha = 0.3,

13. point_size = 2,

14. legend_position = "bottom",

15. color_palette = NULL,

16. add_grid_lines = FALSE,

17. color_points = "primary",

18. fill_voronoi = "primary",,

19. point_shape = "none",

20. label_fontface = "plain",

21. label_size = 3.88,

22. show_island_count = FALSE,

23. label_islands_only = FALSE

24.  )
